# Evaluation of a national smoke-free prisons policy using medication dispensing: an interrupted time-series analysis

**DOI:** 10.1016/S2468-2667(21)00163-8

**Published:** 2021-09-17

**Authors:** Emily J Tweed, Daniel F Mackay, Kathleen A Boyd, Ashley Brown, Thomas Byrne, Philip Conaglen, Peter Craig, Evangelia Demou, Lesley Graham, Alastair H Leyland, Nicola McMeekin, Jill P Pell, Helen Sweeting, Kate Hunt

**Affiliations:** a Medical Research Council/Chief Scientist Office Social and Public Health Sciences Unit, Institute of Health and Wellbeing, University of Glasgow, Glasgow, UK; b Department of Public Health, Institute of Health and Wellbeing, University of Glasgow, Glasgow, UK; c Health Economics and Health Technology Assessment, Institute of Health and Wellbeing, University of Glasgow, Glasgow, UK; d Institute for Social Marketing and Health, University of Stirling, Stirling, UK; e NHS Healthcare Improvement Scotland, Glasgow, UK; f Department of Public Health and Health Policy, NHS Lothian, Edinburgh, UK; g Public Health Scotland, Edinburgh, UK

## Abstract

**Background:**

Internationally, smoking prevalence among people in prison custody (ie, people on remand awaiting trial, awaiting sentencing, or serving a custodial sentence) is high. In Scotland, all prisons implemented a comprehensive smoke-free policy in 2018 after a 16-month anticipatory period. In this study, we aimed to use data on medication dispensing to assess the impact of this policy on cessation support, health outcomes, and potential unintended consequences among people in prison custody.

**Methods:**

We did an interrupted time-series analysis using dispensing data for 44 660 individuals incarcerated in 14 closed prisons in Scotland between March 30, 2014, and Nov 30, 2019. We estimated changes in dispensing rates associated with the policy announcement (July 17, 2017) and full implementation (Nov 30, 2018) using seasonal autoregressive integrated moving average models. Medication categories of primary interest were treatments for nicotine dependence (as an indicator of smoking cessation or abstinence attempts), acute smoking-associated illnesses, and mental health (antidepressants). We included antiepileptic medications as a negative control.

**Findings:**

A 44% step increase in dispensing of treatments for nicotine dependence was observed at implementation (2250 items per 1000 people in custody per fortnight, 95% CI 1875 to 2624) due primarily to a 42% increase in dispensing of nicotine replacement therapy (2109 items per 1000 people in custody per fortnight, 1701 to 2516). A 9% step decrease in dispensing for smoking-related illnesses was observed at implementation, largely accounted for by respiratory medications (−646 items per 1000 people in custody per fortnight, −1111 to −181). No changes associated with announcement or implementation were observed for mental health dispensing or antiepileptic medications (control).

**Interpretation:**

Smoke-free prison policies might improve respiratory health among people in custody and encourage smoking abstinence or cessation without apparent short-term adverse effects on mental health dispensing.

**Funding:**

National Institute of Health Research Public Health Research programme, Scottish Government Chief Scientist Office, and UK Medical Research Council.

## Introduction

In most countries worldwide, the prevalence of tobacco smoking among people in custody is high, in contrast to the decrease observed in the general population.^1^ For example, in Scotland in 2017, 68% of people in prison custody (refers to people on remand awaiting trial, awaiting sentencing, or serving a custodial sentence hereafter) were smokers compared with 18% of adults at liberty,^2^, ^3^ and levels of second-hand smoke in prisons were comparable to those within a typical smoking home.^4^

Smoke-free policies in public places have resulted in substantial reductions in diseases associated with smoking and respiratory, irritant, and sensory symptoms.^5^ However, national smoking bans vary in whether they encompass custodial settings. In the UK, prisons were partially exempt from the 2006–07 legislation on smoke-free enclosed public places; in Scotland people in custody were permitted to smoke in their cells and during outdoor recreation.^6^

Although several jurisdictions worldwide have introduced smoke-free prison policies,^7^ little evidence is available on the health impacts of such policies, particularly with regard to objective measures of health and health-care utilisation. A 2016 Cochrane review identified a need for more robust studies assessing the health impacts of smoking bans in institutional settings such as prisons, including both pre-ban and post-ban data and follow-up for longer than 6 months.^8^

In July, 2017, the Scottish Prison Service announced plans to implement a comprehensive smoke-free policy in the 15 prisons in their estate.^4^, ^9^ This policy, which was implemented on Nov 30, 2018, prohibited smoking in all indoor and outdoor areas and was accompanied by high compliance and immediate, substantial improvements in indoor air quality.^10^

The Tobacco in Prisons study (TIPs) is a multi-method study with a natural experimental design, which has investigated the process and impacts of this policy, using objective air quality measurement; routinely collected data; surveys, interviews, and focus groups with staff and people in custody; and health economic analyses.^11^ In this study, as part of TIPs, we assessed the impacts of this smoke-free policy on treatment for nicotine dependence (as a proxy for smoking cessation or abstinence attempts); specific smoking-associated illnesses; and mental health among people in custody, using routinely collected pharmacy data on medication dispensing in Scottish prisons.

**Research in context**

**Evidence before this study**

Previous studies indicate that smoke-free policies in public places are associated with reductions in acute coronary syndromes, respiratory disease, and sensory symptoms. However, evidence of the health impacts of smoke-free policies in prisons and other institutional settings is scarce. We searched MEDLINE and Embase from database inception to Jan 5, 2021, for published studies on the impact of smoke-free policies in prisons, using synonyms for smoking restrictions ([“smok*” OR “tobacco”] AND [“ban” OR “prohib*”]) combined with those for custodial settings (“prison*” OR “incarcer*”)); and separately for studies on medication usage in relation to smoke-free policies in any context using synonyms for smoking restrictions combined with either terms for medication use (“prescrib*” OR “dispens*”) or for the specific conditions and medications of interest in this study.

Our search yielded 2608 studies. Studies in community settings have found significant associations between ambient air quality and medication dispensing for respiratory conditions, suggesting that medication dispensing might be a valid and sensitive indicator of acute health impacts. Previous research suggests potential reductions in mortality and acute myocardial infarction, and improvements in self-reported health, but might be biased by secular trends, seasonality, or changes in exposure or outcome measurement. Previous systematic reviews have identified a need for high-quality studies assessing the health impact of smoking bans in institutional settings such as prisons. To our knowledge, no previous studies have investigated objective indicators of health impacts among people in custody (including potential unintended harms) using robust designs able to account for underlying trends.

**Added value of this study**

Using routine medication dispensing data for 44 660 people in custody in Scottish prisons (regardless of custodial status) during a 5·7-year period, we found that the implementation of a comprehensive smoke-free policy was associated with a substantial increase in indicators of smoking cessation or abstinence attempts and improvements in indicators of respiratory health, with no evidence of changes in dispensing for mental health. In contrast to previous work in this area, our analyses accounted for underlying trends, seasonal effects, and autocorrelation, with our main findings evident in both modelling strategies used (seasonal autoregressive integrated moving average with prespecified breakpoints and indicator saturation with model-identified breakpoints). We found no change in dispensing rates for antiepileptic medications (control) in response to policy announcement or implementation, which strengthens our confidence in the potentially causal relationship between policy implementation and dispensing rates. This analysis is part of the first study internationally to assess the implementation of a comprehensive smoke-free policy across an entire prison system and, to our knowledge, represents the first use of medication dispensing to assess the impact of smoke-free policies in institutional settings on smoking-related health conditions and mental health.

**Implications of all the available evidence**

This study corroborates existing evidence from community settings that smoke-free policies can result in rapid and sustained improvements in respiratory health and extends this finding to institutional settings largely exempt from the UK smoke-free policy introduced in 2006–07 in public places. Findings are pertinent for other jurisdictions considering smoke-free prison policies. We found that a smoke-free policy had no apparent effect on antidepressant dispensing, which is reassuring with regard to potential unintended consequences for mental health, but does not exclude the possibility of potential negative impacts for some people who are in custody, especially among those most at risk of poor mental health. Medication dispensing seems to be a sensitive and widely available outcome indicator for monitoring population health impacts of tobacco control policies and air quality changes, especially for relatively mild symptoms that might not otherwise result in health-care utilisation, but which collectively could represent a substantial population burden.

## Methods

### Study design

We used an interrupted time-series analysis to quantify changes in medication dispensing in Scottish prisons after the announcement of a smoke-free prisons policy and subsequent implementation of the policy. The population of interest comprised people in custody in Scottish prisons during the analysis period (March 30, 2014, to Nov 30, 2019). Primary analyses included all 14 closed prisons; secondary analyses also included Scotland's one open prison. Details of the Scottish prison estate and population are available online.

We obtained anonymised individual-level dispensing data for people in custody from the single pharmacy provider, which manages procurement and reimbursement of medications for Scottish prisons, via National Health Service (NHS) National Services Scotland. These data were based on individual patient medication records and stock (bulk) supply to prisons, and comprise all medications dispensed in Scottish prisons during the study period with the exception of nicotine replacement therapy in one prison, which is managed by an in-reach service provided by the local health board. This prison was therefore excluded from analyses of nicotine replacement therapy but included in all other analyses.

Prison population data and contracted capacity for the study period were obtained from the Scottish Prison Service, based on the twice-weekly prison census. Prison population data were averaged to obtain a fortnightly mean population.

**Figure 1:**
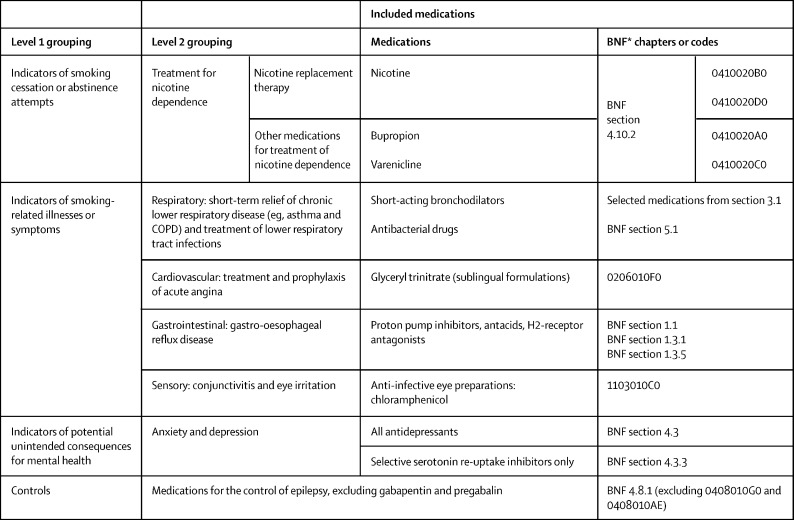
Medications of interest included in the analysis and their grouping for analytical purposes BNF=British National Formulary. COPD=chronic obstructive pulmonary disease. *The BNF is the pharmaceutical reference book used in the UK National Health Service.

Mean dispensing rates per person for each medication category were calculated by dividing the sum of dispensed items by the mean prison population for each fortnightly unit (figure 1). Data cleaning techniques are described in detail in the appendix (p 1).

The TIPs study protocol was approved by the Scottish Prison Service Research Access and Ethics committee and University of Glasgow ethics committee. The analysis reported here used only de-identified dispensing records and aggregate prison population data, collected as part of routine health-care and prison service provision; thus the requirement for written informed consent was waived.

### Outcomes

Selection of medication categories as outcomes was based on an a priori set of criteria informed by the clinical and operational expertise of the co-investigators (appendix p 2). The key outcomes of interest were dispensing rates of medications for treatment of nicotine dependence (as a proxy for smoking cessation or abstinence attempts); for acute smoking-related conditions of the respiratory, cardiovascular, gastrointestinal and sensory systems; and antidepressants. The smoking-associated illnesses category included two categories strongly associated with tobacco smoking and second-hand smoke exposure (respiratory and cardiovascular) and two categories with a weaker association (gastrointestinal and sensory). Since data were not available on the conditions drugs were dispensed for, and a range of antibacterial drugs are used to treat lower respiratory tract infections, we chose to include all antibacterial drugs within the respiratory category. Since antidepressant medications might be used for indications other than mental health (eg, some tricyclic antidepressants are used for neuropathic pain), and in the absence of data on indication, we included a subgroup analysis for selective serotonin reuptake inhibitors (SSRIs) only, because this antidepressant class is most specific to mental health problems. Our original analysis plan did not include hypnotic and anxiolytic drugs in the mental health category due to the widespread use of benzodiazepines for alcohol detoxification in the prison setting; thus we did a post-hoc analysis of this category (appendix p 10). The rationale for not including opioid substitution therapy dispensing as a potential outcome category is included in the appendix (p 2).

We also did an analysis of a control group of medications, expected to be unaffected by the intervention, to address the potential for time-varying confounding by changes in dispensing practice or coding, changes in composition of the population being studied, or co-occurring interventions (table). We chose medications for managing epilepsy as the control, because neither smoking status nor second-hand smoke exposure are known to affect epilepsy onset or severity or the pharmacokinetics of these medications, and dispensing rates were likely to be high enough to provide sufficient statistical power. Gabapentin and pregabalin were excluded from the control group due to their reclassification as class C controlled substances under the Misuse of Drugs Act 1971 during the study period, in October, 2018, and their potential for misuse (which might have resulted in displacement use following the withdrawal of tobacco).

**Table:** SARIMA modelling of changes in fortnightly dispensing rates per 1000 people in custody at smoke-free policy announcement and policy implementation for closed prisons in Scotland

	**Announcement (July 17, 2017)**				**Implementation (Nov 30, 2018)**			
	Step		Slope		Step		Slope	
	Coefficient (95% CI)	p value	Coefficient (95% CI)	p value	Coefficient (95% CI)	p value	Coefficient (95% CI)	p value
**Treatment for nicotine dependence**								
All*	−204·8 (−1564·7 to 1155·2)	0·768	12·6 (−28·1 to 53·4)	0·544	2249·6 (1874·9 to 2624·4)	<0·0001	−2·6 (−44·4 to 39·1)	0·901
Nicotine replacement therapy*	−256·8 (−1244·6 to 731·0)	0·610	16·0 (−13·2 to 45·1)	0·283	2108·6 (1701·3 to 2515·9)	<0·0001	1·1 (−26·1 to 28·3)	0·936
Other (varenicline or bupropion)†	−4·9 (−46·3 to 36·5)	0·817	0·1 (−2·1 to 2·3)	0·953	48·2 (20·0 to 76·5)	0·0008	−9·1 (−14·4 to −3·8)	0·001
**Smoking-associated illness**								
All	−76·4 (−458·9 to 306·1)	0·695	−23·9 (−41·4 to −6·4)	0·007	−646·2 (−1110·9 to −181·4)	0·006	16·5 (−13·6 to 46·7)	0·282
Respiratory	65·7 (−130·4 to 261·9)	0·511	−4·3 (−13·0 to 4·4)	0·330	−485·9 (−746·7 to −225·1)	0·0003	−11·0 (−24·7 to 2·7)	0·114
Cardiovascular	−105·6 (−191·4 to −19·8)	0·016	1·3 (−1·9 to 4·5)	0·418	−49·6 (−170·8 to 71·6)	0·422	−2·2 (−9·4 to 5·1)	0·561
Gastrointestinal	−73·9 (−303·7 to 156·0)	0·529	−21·0 (−32·3 to −9·7)	<0·0001	−137·3 (−507·6 to 233·1)	0·468	30·2 (8·6 to 51·7)	0·006
Sensory	1·4 (0·1 to 2·6)	0·032	−0·1 (−0·2 to 0·0)	0·002	−0·8 (−3·5 − 1·8)	0·541	0·2 (0·0 to 0·3)	0·017
**Mental health**								
All antidepressants†	−119·7 (−270·2 to 30·7)	0·119	−2·9 (−11·5 to 5·6)	0·502	151·7 (−114·2 to 417·5)	0·263	0·5 (−12·6 to 13·6)	0·940
SSRI antidepressants†	−121·3 (−171·9 to −70·7)	<0·0001	−3·1 (−5·7 to −0·5)	0·020	18·2 (−52·0 to 88·4)	0·611	3·2 (−0·1 to 6·4)	0·054
**Control series**								
Antiepileptic drugs†	−19·0 (−94·8 to 56·8)	0·623	2·1 (−1·3 to 5·5)	0·230	−70·9 (−184·8 to 43·1)	0·223	−0·7 (−6·6 to 5·2)	0·820

SARIMA=seasonal auto-regressive integrated moving average. SSRI=selective serotonin reuptake inhibitor.

* Analyses of nicotine replacement therapy and the nicotine replacement therapy component of the combined nicotine dependence category excluded one closed prison for which nicotine replacement therapy was dispensed via an in-reach service provided by the local health board, rather than the national pharmacy contract, and for which detailed data on nicotine replacement therapy dispensing were therefore not available; data for this prison were included in all other analyses.

† SARIMA models provided the best model fit for these outcomes considering seasonality in dispensing associated with the Christmas period.

We considered and rejected the possibility of using medication dispensing among the non-prison population of Scotland as a control series, due to differences in population characteristics and co-occurring interventions that were likely to undermine the strength of the counterfactual, and pragmatic challenges in obtaining national community dispensing data.

### Statistical analysis

To distinguish the effects of policy announcement and implementation, we divided the analysis period (March 30, 2014, to Nov 30, 2019) into three phases: pre-announcement (March 30, 2014, to July 17, 2017); anticipatory (July 18, 2017, to Nov 29, 2018); and post-implementation (Nov 30, 2018, to Nov 30, 2019). The design and analysis were prespecified in a published protocol.^12^ Changes to the protocol are described in the appendix (p 4).

We analysed the data using auto-regressive integrated moving average (ARIMA) models, including seasonal ARIMA (SARIMA) models where appropriate, to account for underlying secular trends, seasonality, and autocorrelation. We modelled the coefficients for step and slope changes in dispensing rates at the transition points between the pre-announcement, anticipatory, and post-implementation phases, using indicator variables reflecting the dates of policy announcement and implementation. Since overcrowding is acknowledged as an important determinant of health in the prison setting, a crowding indicator for use as a covariate in sensitivity analyses was calculated on the basis of the ratio of the observed fortnightly mean population to the contracted capacity (ie, the number of people in custody that the prison is contracted by the Scottish Prison Service to hold) of the prison estate recorded for that period. The choice of model was based on the Box-Jenkins three-step approach of identification of auto-regressive and moving average components, using autocorrelation and partial autocorrelation functions; model estimation; and diagnostic checking, using the Portmanteau Q statistic for white noise residuals, kernel density plots to assess normality of residuals, and the Akaike Information Criterion and Bayesian Information Criterion for each model.^13^ We first modelled the whole time series before modelling and testing the effect of policy announcement and implementation (appendix p 6).

The impact of the smoke-free policy was hypothesised to differ in important ways in open versus closed establishments, because people in custody in Scotland's open prison might smoke on periods of home leave, or while working outside of the prison. Primary analyses therefore comprised fortnightly dispensing rates for the 14 closed prisons in Scotland. For primary analyses, we additionally calculated relative effect estimates for step and slope changes by applying the absolute coefficients for the change obtained from ARIMA or SARIMA modelling to mean dispensing rates immediately preceding announcement and implementation (appendix p 7).

We did prespecified secondary analyses comprising: all 15 prisons in Scotland (open and closed); indicator saturation to identify step or slope changes not specified a priori;^14^ weekly time series; and adjustment for the crowding indicator. Since qualitative analyses of TIPs suggested some stockpiled tobacco might have been in circulation immediately after implementation of the smoke-free policy, we did a post-protocol analysis to test whether specifying a later implementation date (Dec 30, 2018) provided a better model fit. Full results of secondary and post-protocol analyses are in the appendix (pp 7–14).

Data cleaning and ARIMA or SARIMA modelling were done using Stata software (version 16); indicator saturation analyses were done using R software (version 3.6.3) using the *gets* package.

### Role of the funding source

The funders of the study had no role in study design, data collection, data analysis, data interpretation, or writing of the report**.**

## Results

The mean daily prison population during the study period was 7517 (SD 235·4, range 6984–8143) for the closed estate (primary analyses) and 7730 (235·4, 7185–8335) for all prisons (secondary analyses). 44 660 unique individuals were estimated to have spent time incarcerated in the closed estate in Scotland during the study period (primary analyses); and 44 775 individuals for all prisons (secondary analyses). A total of 148 fortnights (86 pre-announcement, 35 in the anticipatory period, and 27 post-implementation) and 31·3 million eligible dispensed items; 3 324 178 items for nicotine dependence, 16 850 875 items for smoking-associated illnesses, 9 214 162 items for mental health, 1 885 990 for the control condition) were included in the analyses. Total dispensed quantities and mean rates for each medication category during the overall study period and each phase are shown in the appendix (p 5).

**Figure 2:**
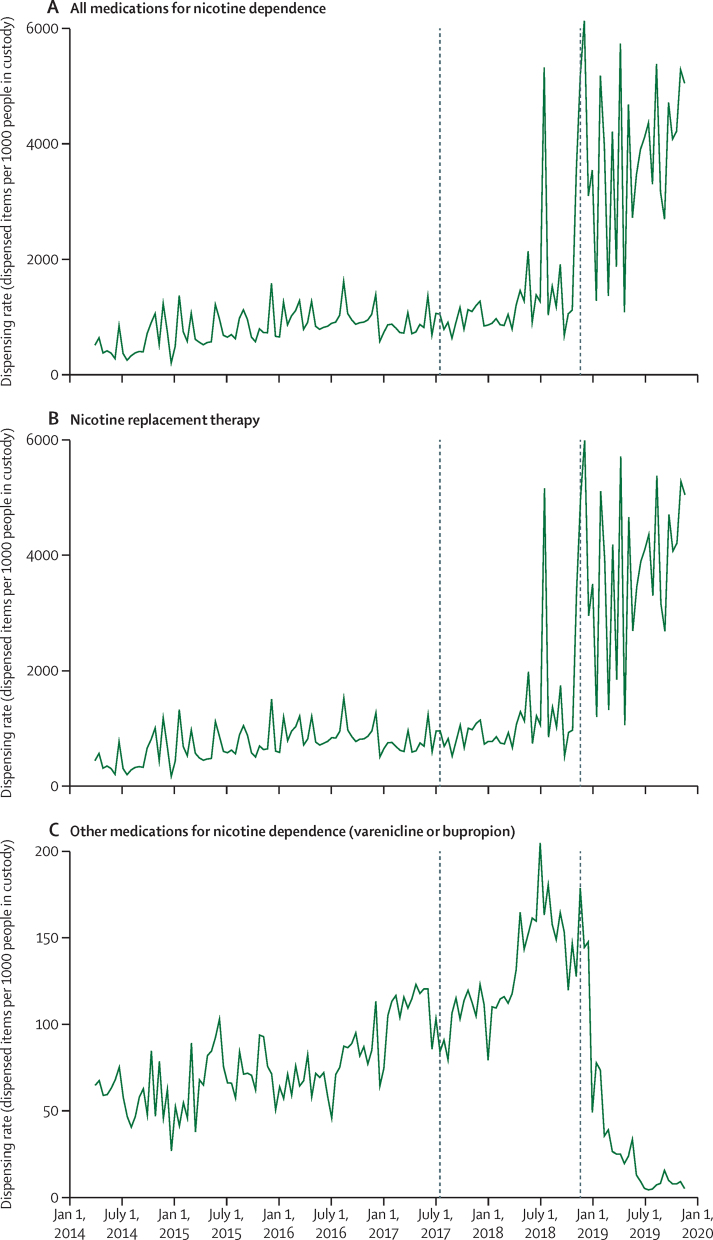
Time-series analysis of fortnightly dispensing rate for medications for nicotine dependence in Scottish prisons during the study period Dashed lines indicate policy announcement and implementation dates.

Within the overall category of medications for nicotine dependence, a 44% increase in dispensing was observed (figure 2A, table; appendix p 11), primarily driven by nicotine replacement therapy, which accounts for the majority of dispensing in this category. For nicotine replacement therapy, a 42% increase in dispensing associated with policy implementation was observed (2109 items per 1000 people in custody per fortnight, 95% CI 1701–2516; figure 2B, table; appendix p 11).

An initial step increase in dispensing rates of other medications for nicotine dependence was observed (48 items per 1000 people in custody per fortnight, 95% CI 20 to 77) with a negative slope change (−9 items, 95% CI −14 to −4) at the point of policy implementation (figure 2C, table). Sensitivity analysis demonstrated that a delayed implementation date (Dec 30, 2018) provided a better fit to the data on dispensing of other medications for nicotine dependence, indicating that an initial peak immediately after implementation was followed by a sustained step decrease in dispensing (−80 items per 1000 people in custody per fortnight, 95% CI −106 to −53; appendix p 7) and a negative slope trend (−5 items, 95% CI −2 to −8).

**Figure 3:**
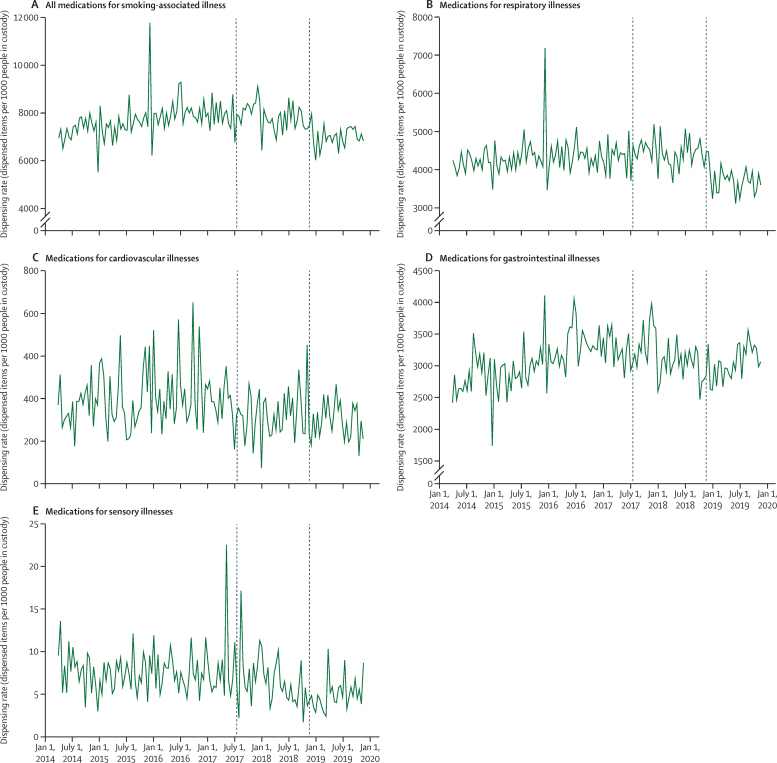
Time-series analysis of fortnightly dispensing rate for medications for smoking-associated illnesses in Scottish prisons during the study period (A) All medications for smoking-associated illnesses. (B) Medications for respiratory illnesses. (C) Medications for cardiovascular illnesses. (D) Medications for gastrointestinal illnesses. (E) Medications for sensory illnesses. Dashed lines indicate policy announcement and implementation dates.

For medications for smoking-related illnesses, primary analysis of the combined category suggested a negative slope change at the point of policy announcement (−24 items, 95% CI −41 to −6; 0·3% relative decrease) followed by a step decrease on implementation (−646 items per 1000 people in custody per fortnight, 95% CI −1111 to −181; 9% relative decrease; figure 3A, table; appendix p 11).

For respiratory disease, a substantial step decrease was observed at the point of policy implementation (−486 items per 1000 people in custody per fortnight, 95% CI −747 to −225; 11% relative decrease), which largely accounted for the observed decrease in overall smoking-related illness dispensing (figure 3B, table).

For cardiovascular conditions, a substantial step decrease was observed at the point of policy announcement (figure 3C, table), although the 95% CIs were wide (−106 items per 1000 people in custody per fortnight, 95% CI −191 to −20; 44% relative decrease). No other significant changes in dispensing for cardiovascular conditions were observed.

For gastrointestinal diseases, there was a pre-existing upward trend in dispensing rates, which plateaued at the point of policy announcement and subsequently resumed at the point of implementation (figure 3D, table). For the sensory disease category, dispensing rates were low overall (figure 3E). Modelling suggested a similar pattern to gastrointestinal diseases, with a small step increase observed at announcement, but the absolute changes were small (around 1 or fewer items per 1000 people in custody per fortnight; 0·1–0·2% relative change) and most confidence intervals included zero (table).

**Figure 4:**
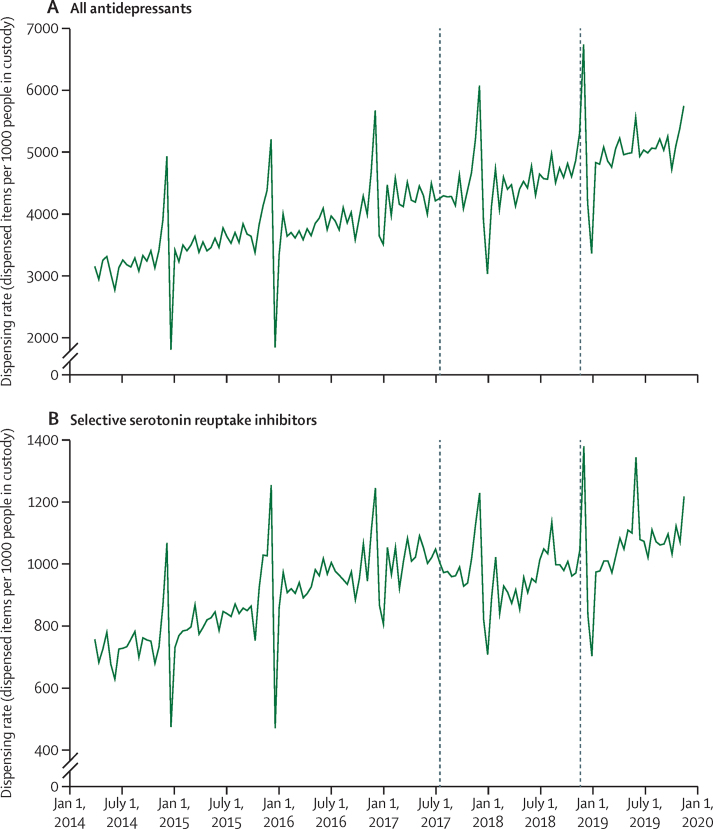
Time-series analysis of fortnightly dispensing rate for antidepressant medications in Scottish prisons during the study period Dashed lines indicate policy announcement and implementation dates.

No significant changes in dispensing rates of antidepressant medications were observed in association with policy announcement or implementation in either primary (figure 4A, table) or sensitivity analyses (appendix p 7). For the subgroup of SSRI antidepressants, policy announcement was associated with a negative step change (−121 items per 1000 people in custody per fortnight, 95% CI −172 to −71; 12% relative decrease) and small negative slope change (−3 items, 95% CI −6 to 0; 0·1% relative decrease), followed by a positive slope change of similar magnitude at the point of policy implementation (3 items, 95% CI 0 to 6; figure 4B, table).

**Figure 5:**
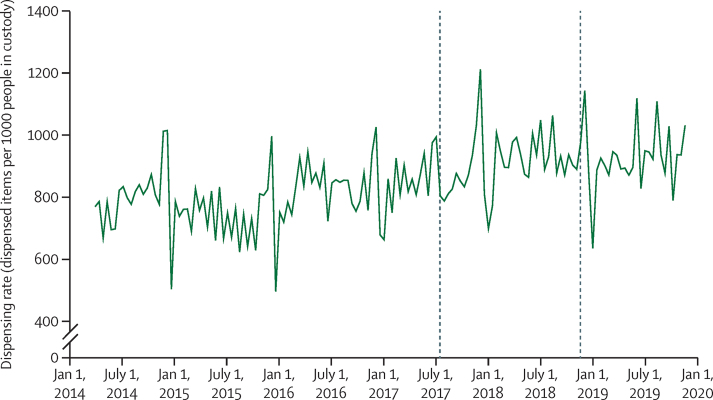
Time-series analysis of fortnightly dispensing rate for antiepileptic medications in Scottish prisons during the study period Dashed lines indicate policy announcement and implementation dates.

No significant changes in the dispensing rates of antiepileptic medications (control) were observed at announcement or implementation (figure 5, table).

Indicator saturation results for nicotine replacement therapy, other medications for nicotine dependence, and respiratory medications confirmed substantial changes following policy implementation, with dates in December, 2018 (2–4 weeks after official implementation), identified as the key breakpoint for step changes (appendix p 12). For other outcomes, no significant changes associated with policy announcement or implementation were identified. The modest step and slope changes in dispensing rates for non-respiratory smoking-associated illnesses observed in primary analyses were not confirmed by indicator saturation. Results of sensitivity analyses were otherwise consistent with the primary results, including those encompassing the open prison (appendix p 7). A post-hoc analysis of dispensing of hypnotic or anxiolytic medications did not identify any changes associated with policy announcement or implementation (appendix p 7).

## Discussion

Implementation of a comprehensive smoke-free policy in Scottish prisons was associated with increased overall provision of treatment to support smoking cessation or abstinence, reflecting a substantial increase in the dispensing of nicotine replacement therapy and a smaller decline in dispensing of other medications (primarily varenicline) in this category. Implementation was also associated with a sustained reduction in dispensing of medications for respiratory illness. A modest reduction in dispensing for acute angina at the point of announcement and temporary changes in dispensing for sensory and gastrointestinal symptoms were not corroborated in sensitivity analyses using an alternative modelling approach. We found no evidence of increased dispensing for depression and anxiety, and, when examining medications most specific to these indications, a suggestion of short-term reductions in dispensing.

To our knowledge, this is the first analysis to use medication dispensing to assess the impact of smoke-free policies in institutional settings on outcomes other than smoking cessation or abstinence attempts, and forms part of the first study internationally to assess implementation of a comprehensive smoke-free policy across an entire prison system.

Previous studies have reported changes in prescribing of nicotine replacement therapy following smoke-free policies in psychiatric hospitals, although these were simple comparisons done before and after policy implementation that did not account for secular trends, seasonality, or autocorrelation.^15^, ^16^, ^17^ Our observation of increased nicotine replacement therapy dispensing is consistent with these studies and findings from the qualitative data collected as part of TIPs, which described extensive preparations across the Scottish Prison Service for increased demand for cessation or abstinence support.^11^ The observed decline in dispensing of other medications used to support cessation or abstinence attempts might be explained by multiple factors, including a decline in opportunities to prescribe varenicline for the licensed indication of cessation of tobacco smoking; the increased availability of nicotine replacement therapy, which is recommended as first line in local formularies in Scotland and is substantially cheaper than other medications used to support cessation or abstinence attempts; and widespread availability and uptake of rechargeable e-cigarette devices.^18^

Our finding of decreased dispensing for respiratory conditions following policy implementation is consistent with studies of the association between ambient air quality and medication dispensing, which found a consistent positive association between dispensing for chronic respiratory conditions and airborne pollutant levels,^19^, ^20^, ^21^ and with observed reductions in respiratory symptoms and hospital admissions after community smoke-free legislation.^22^ By documenting the impact of a clearly defined intervention in a closed setting with substantial improvement in measured air quality,^10^ our study addresses some limitations of existing community-based outcome studies, for which exposure measurement is more challenging.^21^

Fewer studies have investigated the association between air quality (indoor or outdoor) and dispensing for non-respiratory outcomes than for respiratory outcomes.^23^ We found a potential modest impact of policy announcement on dispensing for acute angina, and no clear effect in either direction for dispensing for gastro-oesophageal reflux disease and conjunctivitis, none of which were replicated in secondary analyses using indicator saturation (ie, where dates of interest are identified by testing for potential breaks at every point in the time series rather than prespecified). These findings might reflect low rates of dispensing (for cardiovascular and sensory illnesses) or weaker associations with first-hand or second-hand smoke exposure (for gastrointestinal and sensory illnesses) for these outcomes. The inconclusive results for cardiovascular dispensing contrast with the strong evidence of a reduction in cardiovascular events following smoking bans in community settings^24^ and might reflect the relatively young population in Scottish prisons (mean age 36 years in 2019–20),^25^ although this population are at risk of accelerated onset of long-term conditions and multimorbidity.^26^ Long-term studies of cardiovascular impacts of smoke-free policies, especially among older people in custody and those serving longer sentences, are warranted.

Our findings on smoking-associated illness among people in custody are corroborated by a parallel arm of TIPs, which reported reductions in recorded staff sickness absence overall and for cardiothoracic conditions after policy implementation.^11^ The absence of changes in antidepressant or hypnotic or anxiolytic drug dispensing among people in custody is reassuring, especially considering the broader evidence base that smoking cessation can have mental health benefits,^27^ but should be interpreted with caution since medication dispensing is a crude indicator of potential mental health impacts and might not capture heterogeneous effects—eg, among people in custody at high risk of poor mental health. In TIPs surveys, two-thirds of people in custody reported that the smoking ban had made them more anxious and only 12% agreed it had made them happier, although response rates were low.^11^

A major strength of this study is its national coverage, with comprehensive outcome data covering almost all dispensing episodes in Scottish prisons during the study period (with the exception of nicotine replacement therapy for one prison, which was omitted from analyses for that outcome). Our time-series analysis used data collected over a longer duration than most similar studies,^8^, ^28^ which increases the likelihood of detecting subtle effects and adequately accounting for seasonal and secular trends. Our use of pharmacy contract data collected for the purposes of financial reimbursement is likely to maximise validity in terms of data quality and completeness. Use of a consistent single source of administrative data for outcome measurement throughout the time series also helps mitigate against artifactual changes over time (instrumentation bias). The absence of effect in a control series of medications increases our confidence in inferring a causal relationship between the smoke-free policy and observed changes in dispensing.^28^ We were not able to include a control series of prisons where the intervention was not implemented, since the policy was introduced in all Scottish prisons simultaneously, or to investigate heterogeneity of impacts within the prison population. We also were unable to include data on rechargeable e-cigarette use, which might have affected dispensing to support smoking cessation or abstinence following their introduction 2 months before implementation of the smoke-free policy.^11^

Medication dispensing has not, to our knowledge, previously been used to investigate smoke-free policies in custodial settings, although it has been extensively used as an outcome in studies of the health impacts of outdoor air quality^21^ and as part of a composite outcome for monitoring respiratory symptoms following implementation of community smoke-free policies in one study.^29^ The use of medication dispensing as a proxy indicator of health impacts has advantages and limitations. Medication dispensing is an objective indicator of health impacts that avoids response bias and captures relatively mild conditions or those usually managed in primary care, which might have substantial human and economic costs. Most other studies of public or institutional smoke-free policies have focused on hospital admissions or mortality, which represent only one component of the broader health impact of smoke-free policies. Although dispensing might not always reflect use, it does reflect demand and thus is a reasonable proxy for symptoms: this might be especially true in closed settings such as prisons. However, some of our medication categories, such as antibiotics and antidepressants, were non-specific for the outcomes of interest because data on reason for dispensing were not available: this might have resulted in bias towards the null for relative effect estimates, although this should not have affected estimates of absolute changes. In particular, antidepressant dispensing is a crude indicator of consequences for mental health, and data from the survey and qualitative components of the TIPs suggest that these impacts should not be overlooked.^11^

Our results suggest that smoke-free prison policies have beneficial effects on acute respiratory illness during imprisonment. However, since most people in prison globally are in pre-trial detention or serving short sentences, with the average time served by people in prison custody in Scotland less than 6 months,^25^ the long-term impact of such policies will depend on whether these policies encourage sustained abstinence from smoking outside the prison environment. Evidence to date suggests that smoking relapse rates are high after release from smoke-free prisons, although such policies might reduce the intensity of smoking after release.^30^ Prison smoke-free policies must therefore form part of a comprehensive package of tobacco control measures encompassing both community and custodial settings, and broader efforts to address health inequalities among people who experience incarceration.^31^ In addition to work to quantify and support sustained behavioural change, future studies of smoke-free prison policies should investigate longer-term health impacts among people in custody and staff, perhaps using linkage between prison records and primary or secondary health-care data on diagnoses and hospital admissions.

For more on the **Scottish prison estate and population** see https://www.sps.gov.uk/Corporate/Corporate.aspx

For the **study protocol** see https://osf.io/j8uzd/

For the **TIPs study protocol** see https://fundingawards.nihr.ac.uk/award/15/55/44

## Data sharing

The aggregate dispensing data used in this study are available to researchers on request from NHS National Services Scotland, and the original study protocol is available online.

## Declaration of interests

HS was a member of Action on Smoking and Health (ASH) Scotland's Board (2017–20) and the Policy and Development Committee (2013–20), chair of the Scottish Tobacco-free Alliance Research Group (2012–20), and a council member for the Tobacco-free Alliance (2015–20). KH was previously a member of the National Institute for Health Research (NIHR) Public Health Research funding board.
