# Supplementary material for: Evaluation of a national smoke-free prisons policy using medication dispensing: an interrupted time-series analysis
Source: Lancet Public Health. 2021 Sep 17;6(11):e795–804. doi: 10.1016/S2468-2667(21)00163-8 (PMC8554387; doi:10.1016/S2468-2667(21)00163-8)
Supplement: Supplementary appendix [file mmc1.pdf]

THE LANCET
Public Health

Supplementary appendix

This appendix formed part of the original submission and has been peer reviewed. We post it as supplied by the authors.

Supplement to: Tweed EJ, Mackay DF, Boyd KA, et al. Evaluation of a national smoke-free prisons policy using medication dispensing: an interrupted time-series analysis. *Lancet Public Health* 2021; published online Sept 16. http://dx.doi.org/10.1016/S2468-2667(21)00163-8.

Supplementary material

Section S1. Details of data cleaning methods.

Dispensing data

Dispensing data were supplied by the pharmacy contractor as CSV files covering monthly or quarterly and comprising individual dispensing events. These data were appended together to obtain a dataset covering the study period and subsequently checked for duplicates and missing values. Dispensing dates were converted to week-year and fortnight-year values, with each week or fortnight period beginning on a Sunday. Dates and quantities were checked for implausible values using summary statistics and visual methods.

A consistent set of prison names were derived, correcting for misspellings; case differences; and use of hall names rather than overall institutions. Time series of dispensing quantities and rates were plotted for each prison and overall by daily, weekly, and fortnightly date as well as by day of the week and month of the year in order to check for consistency and any errors or outliers.

Relevant medications were identified by a combination of automatic searching of BNF subchapter and product name fields, and hand-searching of product name field. A manual check of product names included under each category was undertaken to complete this process. Datasets were created for each level 1 and level 2 medication category, containing dispensed quantities overall and by prison, by week-year and fortnight-year, with indicator variables to identify whether each time period fell during the pre-announcement, anticipatory, or post-implementation phase. Separate totals were calculated for the closed prison estate (primary analyses) and the entire prison estate including the one open prison (secondary analyses).

Population data

Population data were supplied by Scottish Prison Service in PDF files which were converted to CSV files using the Tabula (https://tabula.technology/) programme and hand-checked by two authors. These were appended together to obtain a dataset covering the study period and subsequently checked for duplicates and missing values.

Time series of population counts (used as denominators for dispensing rates, and numerators for the crowding indicator variable) and contracted capacity (used as denominators for the crowding indicator variable) were plotted for each prison and overall by daily, weekly, and fortnightly date as well as by day of the week and month of the year in order to check for consistency and any errors or outliers. Separate totals were calculated for the closed prison estate (primary analyses) and the entire prison estate including the one open prison (secondary analyses).

A crowding indicator was calculated using population count divided by contracted capacity, by prison and overall.

Combination of dispensing data and population data to create dispensing rates

Files containing dispensing data and population data were merged and new variables created to calculate dispensing rates per week and per fortnight for each prison, the closed prison estate as a whole (primary analyses), and the entire prison estate including the one open prison (secondary analyses). Times series of mean dispensing rates for each level 1 and level 2 medication category by week and by fortnight were plotted to check for consistency and any errors or outliers.

Section S2.1. Criteria for selection of medication categories as outcomes of interest for evaluating the intended and unintended consequences of the smokefree prison policy

- Indicators of attempts to quit or abstain from smoking, or of a health condition with potential to be affected by one or more of the following:
    - Smoking status;
    - Exposure to SHS;
    - Other, unintended consequences of the policy.
- Unlikely to be confounded by co-occurring changes in demand or dispensing practice
- Responsive to change within the one-year follow up post-intervention
- Likely to be dispensed in quantities sufficient to provide adequate statistical power

Section S2.2. Rationale for not including opioid substitution therapy (OST) as a medication category among the outcomes of interest

In response to reviewer feedback, we include here a detailed rationale for not including OST as a medication category of interest among the study outcomes.

Opioid dependence is a prevalent condition among people in custody and there are potential unintended consequences of smokefree policies on this population with regard to the severity of their dependence and demand for substitution therapies. This might result in a change in the dosage of methadone dispensed for an individual in response to policy implementation, although is unlikely to have a significant effect on the number of individuals with opioid dependence for whom OST is indicated or their motivation for or access to treatment.

However, there are a number of potential issues which complicate the use of OST dispensing as an indicator of unintended consequences. The metabolism – and therefore the serum levels and appropriate dosage – of methadone and other forms of opioid is affected

by exposure to polycyclic aromatic hydrocarbons found in tobacco smoke (see for instance, Wahawisan et al 2011 - https://doi.org/10.1345/aph.1P759). The dramatic reduction in levels of second-hand smoke demonstrated quantitatively in other strands of the TIPs project (e.g. Demou et al 2020 - https://doi.org/10.1093/annweh/wxaa073) means that lower doses may have been required to achieve the same effect among PiC following the intervention. There were also a number of concurrent unrelated trends and events which may affect OST dispensing during the study period, including ongoing public debates about the benefits and risks of OST; concerns about rising drugs deaths; the launch of an updated drug treatment strategy by Scottish Government; and the publication of new UK-wide clinical guidelines on drug treatment, including OST use.

For all these reasons, we decided a priori not to include OST in our planned analyses, prior to reviewing the data or undertaking any analysis. This position was informed by discussions with one of our co-authors (TB) who is National Prisons Pharmacy Adviser for Scotland, who strongly advised against use of the dispensing data for OST as an outcome for the evaluation for the reasons outlined above.

Section S3. Amendments to original protocol and rationale

Amendment	Rationale
Analysis of hospitalisations omitted from project as not feasible due to data quality issues.	Data quality issues with the recording of address details in SMR01 (Scottish Morbidity Record 01; source of data on general and acute inpatient and day case hospital stays in Scotland) meant that our primary approach for identifying people admitted to hospital from custody using prison postcodes was not reliable for the study period of interest. Given that other methods of identifying people admitted to hospital from prison from within the SMR01 dataset itself also had important limitations, and there was insufficient time to undertake individual-level data linkage between prison records and SMR01, we were required to omit the hospitalisations analysis from the project.
Use of fortnightly intervals for time series in primary analyses rather than weekly. Models for weekly data run as a secondary, 'per-protocol' analysis and shown in supplementary material.	On receipt of dispensing data, it was found to show substantial week-to-week variation which complicated the identification of appropriate time series properties. Fortnightly data felt to be more appropriate for identifying underlying time series properties and trends, and modelling intervention.
Study period to start from 30th March 2014 rather than 1st January 2013	2013 and early 2014 saw significant changes to the prison system in north-east Scotland, with the closure of two prisons (HMP Peterhead and HMP Craiginches and the opening of another (HMP Grampian). In order to exclude artefactual changes in dispensing associated with prison closure and opening and ensure a consistent series of prisons included in the analyses, the study period has been amended to begin one month after the opening of HMP Grampian.
Data from one prison on dispensing of nicotine replacement therapy and combined nicotine dependence category omitted from analyses of these outcomes	It was identified after protocol submission that nicotine replacement therapy in this prison is primarily provided by an in-reach service run by the local health board rather than being dispensed via the national pharmacy contract, so will not be captured in our data. In-reach data collected differently and may not be complete, so we decided against including it in the series. Instead, results from this prison are omitted from analyses of relevant outcome categories (nicotine replacement therapy and combined nicotine dependence category).
Restriction of respiratory category to short-acting inhaled bronchodilators and antibiotics; restriction of cardiovascular category to short-acting nitrate formulations	Protocol did not specify whether inhaled bronchodilators (respiratory category) and nitrates (cardiovascular) included both short-acting and long-acting preparations. Protocol also included inhaled corticosteroids, which are used for long-term control of obstructive airways disease.<br><br>Demand for long-acting bronchodilators, inhaled corticosteroids, and long-acting nitrate formulations was felt to be unlikely to change in response to changes in tobacco smoke exposure and therefore less appropriate indicators of intervention effectiveness.<br><br>Prior to data analysis, we therefore decided to omit these medications and restrict analyses to short-acting bronchodilators and antibiotics (respiratory category) and short-acting nitrate formulations (cardiovascular category) only.
Addition of hypnotics/anxiolytics category to mental health analysis in response to reviewer feedback	Request from reviewers to investigate potential changes in this category. Multiple indications for hypnotics/anxiolytics but no major confounding trends during study period identified *a priori*, so analyses undertaken in response to this request.

Section S4. Dispensed quantities and mean dispensing rates per 1000 persons in custody for relevant medication categories in closed prisons during each study phase

	Overall study period (30/03/2014 – 30/11/2019)		Pre-announcement phase (30/03/2014 – 17/07/2017)		Anticipatory phase (18/07/2017 – 29/11/2018)		Post-implementation phase (30/11/2018 – 30/11/2019)	
	Number of dispensed items	Mean dispensing rate per 1000 PiC (95% CI)	Number of dispensed items	Mean dispensing rate per 1000 PiC (95% CI)	Number of dispensed items	Mean dispensing rate per 1000 PiC (95% CI)	Number of dispensed items	Mean dispensing rate per 1000 PiC (95% CI)
**Smoking cessation/abstinence attempts: treatment for nicotine dependence**								
All	3,324,178	1,464.3 (1,242.5 – 1,686.0)	1,012,117	795.5 (731.7 – 859.3)	663,647	1,277.2 (977.6 – 1,576.7)	1,648,414	3,837.0 (3,294.9 – 4,379.1)
Nicotine replacement therapy	3,146,853	1,383.7 (1,160.1 – 1,607.2)	917,080	720.8 (658.4 – 783.1)	597,066	1,148.7 (852.4 – 1,444.9)	1,632,707	3,799.7 (3,259.6 – 4,339.9)
Other (varenicline/bupropion)	177,325	80.6 (73.7 – 87.5)	95,037	74.7 (70.0 - 79.4)	66,581	128.5 (118.3 – 138.7)	15,707	37.2 (18.5 – 56.0)
**Smoking-related illnesses**								
All	16,850,875	7,598.5 (7,481.4 – 7,850.7)	9,794,791	7,688.7 (7,526.7 – 7,850.7)	4,049,281	7,840.1 (7,650.6 – 8,029.6)	3,006,803	6,998.1 (6,823.7 – 7,172.6)
Respiratory	9,395,092	4,238.5 (4,160.7 – 4,316.3)	5,496,581	4,313.7 (4,216.8 – 4,410.6)	2,297,430	4,447.2 (4,326.9 – 4,567.6)	1,601,081	3,728.3 (3,591.6 – 3,865.1)
Cardiovascular	602,035	271.8 (255.9 – 287.7)	380,020	298.6 (276.5 – 320.6)	126,970	245.3 (215.9 – 274.7)	95,045	221.1 (296.3 – 246.0)
Gastrointestinal	6,838,487	3,081.3 (3,023.6 – 3,139.0)	3,908,444	3,068.8 (2,985.4 – 3,152.1)	1,621,528	3,141.1 (3,027.1 – 3,255.1)	1,308,515	3,043.7 (2,940.2 – 3,147.2)
Sensory	15,261	6.9 (6.4 – 7.4)	9,746	7.6 (7.1 – 8.2)	3,353	6.5 (5.5 – 7.5)	2,162	5.9 (5.6 – 6.2)
**Mental health: anti-depressants**								
All	9,214,162	4,139.2 (1,510.4 – 7,683.5)	4,728,469	3,714.8 (3,590.0 – 3,839.6)	2,318,877	4,485.9 (4,327.3 – 4,644.6)	2,166,817	5,041.5 (4,824.2 – 5,258.9)
SSRI anti-depressants	2,073,327	932.3 (908.5 – 956.2)	1,119,881	879.8 (849.2 – 910.5)	498,101	963.6 (931.7 – 995.5)	455,345	1,059.1 (1,008.3 – 1,109.8)
Hypnotics/anxiolytics (post-protocol analysis)	1,823,174	870.7 (799.8 – 841.6)	1,034,614	811.8 (785.5 – 838.2)	430,046	832.1 (779.5 – 884.7)	358,514	834.4 (789.8 – 879.2)
**Control series**								
Anti-epileptics	1,885,990	848.4 (829.6 – 867.2)	1,018,385	799.4 (77.6 – 821.3)	469,112	907.8 (874.6 – 941.0)	398,493	927.3 (886.8 – 967.7)

Section S5. Model specification for primary analyses

	Model specification		
**Outcome of interest**	**AR/MA terms**	**Seasonal terms**	**Any outliers**
All nicotine dependence	AR(1,2,7)	None required	Outlier term for bulk dispensing event in fortnight commencing (f/c) 15/07/2018
Nicotine replacement therapy	AR(1,2)	None required	Outlier term for bulk dispensing event in f/c 15/07/2018
Other medications for nicotine dependence (varenicline/bupropion)	AR(1,2,11)	Seasonal term of AR(1) at interval of one year	Outlier term for bulk dispensing event in f/c 15/07/2018
All smoking-related illness	AR(1,14)	None required	Outlier term for bulk dispensing events in f/c 21/12/2014 & f/c 06/12/2015
Smoking-related illness: respiratory	AR(1,9,14,15,28)	None required	Outlier term for bulk dispensing event in f/c 06/12/2015
Smoking-related illness: cardiovascular	None required	None required	None required
Smoking-related illness: gastrointestinal	None required	None required	None required
Smoking-related illness: sensory	AR(1)	None required	Outlier term for bulk dispensing event in f/c 07/05/2017
Mental health: all anti-depressants	AR(1)	Seasonal term of AR(1) at interval of one year	None required
Mental health: SSRI only	None required	Seasonal term of AR(1) at interval of one year	None required
Mental health: hypnotics/anxiolytics (post-protocol analysis)	None required	Seasonal term of AR(1) at interval of one year	None required
Control: anti-epileptics	AR(2,4)	Seasonal term of AR(1) at interval of one year	None required

.

Section S6. Full results table including secondary and post-protocol analyses: modelling of fortnightly dispensing rates per 1,000 persons in custody

	Announcement				Implementation					
	Step		Slope		Step		Slope		Model fit	
	Coefficient (95% CI)	P value	Coefficient (95% CI)	P value	Coefficient (95% CI)	P value	Coefficient (95% CI)	P value	AIC/ BIC	Adj. R^2
**ALL NICOTINE DEPENDENCE**										
Closed prisons	-204.8 (-1,564.7 – 1,155.2)	0.768	12.6 (-28.1 – 53.4)	0.544	2,249.6 (1,874.9 – 2,624.4)	<0.001	-2.6 (-44.4 – 39.1)	0.901	2337 2370	80.2
All prisons	-132.5 (-1,388.7 – 1,123.7)	0.836	10.7 (-35.0 – 56.3)	0.647	2443.8 (2,053.3 – 2,834.2)	<0.001	-6.7 (-45.0 – 31.6)	0.731	2323 2356	82.3
Adjusted for crowding	-219.5 (-1,570.5 – 1,131.6)	0.750	14.9 (-32.8 – 62.7)	0.540	2255.1 (1,880.9 – 2,629.2)	<0.001	-1.7 (-43.1 – 39.6)	0.934	2339 2375	80.3
Delayed implementation	-762.2 (-2,009.9 – 485.4)	0.231	61.9 (23.1 – 100.7)	0.002	-359.6 (-874.2 – 154.9)	0.171	30.5 (-16.9 – 77.9)	0.207	2372 2399	74.7
Weekly intervals	-401.0 (-976.1 – 174.0)	0.172	11.9 (2.9 – 20.9)	0.010	1,817.8 (1,475.1 - 2,160.6)	<0.001	-5.1 (-14.6 – 4.4)	0.289	4957 5020	68.0
**NICOTINE REPLACEMENT THERAPY**										
Closed prisons	-256.8 (-1,244.6 – 731.0)	0.610	16.0 (-13.2 – 45.1)	0.283	2,108.6 (1,701.3 – 2,515.9)	<0.001	1.1 (-26.1 – 28.3)	0.936	2336 2369	80.1
All prisons	-161.1 (-1,660.2 – 1,338.0)	0.833	9.9 (-39.8 – 59.6)	0.695	2,410.2 (2,008.9 – 2,811.5)	<0.001	7.1 (-39.0 – 53.2)	0.763	2323 2353	82.2
Adjusted for crowding	-269.5 (-1,502.2 – 963.2)	0.668	17.1 (-26.1 – 60.2)	0.438	2,141.9 (1,736.1 – 2,547.8)	<0.001	8.3 (-28.7 – 45.3)	0.661	2340 2373	80.2
Delayed implementation	-750.1 (-2,018.7 – 518.5)	0.247	61.2 (19.5 – 102.9)	0.004	-314.5 (-847.4 – 218.5)	0.247	37.2 (-14.1 – 88.4)	0.155	2373 2403	74.8
Weekly intervals[1]	-298.3 (-1,196.8 – 600.2)	0.515	11.1 (-3.8 – 26.1)	0.144	1,889.5 (1,489.9 – 2,289.2)	<0.001	-1.8 (-13.6 – 10.0)	0.762	4977 5047	66.7
**OTHER MEDICATIONS FOR NICOTINE DEPENDENCE (VARENICLINE/BUPROPION)**										
Closed prisons	-4.9 (-46.3 – 36.5)	0.817	0.1 (-2.1 – 2.3)	0.953	48.2 (20.0 – 76.5)	0.001	-9.1 (-14.4 – -3.8)	0.001	1244 1280	87.5
All prisons	-7.3 (-66.6 – 52.0)	0.809	0.0 (-2.6 – 2.6)	0.997	29.1 (4.9 – 53.3)	0.018	-7.4 (-11.6 – -3.2)	0.001	1288 1321	82.7
Adjusted for crowding	-4.1 (-46.9 – 38.6)	0.850	-0.3 (-2.7 – 2.0)	0.782	49.8 (12.8 – 86.8)	0.008	-9.6 (-15.8 – - 3.5)	0.002	1242 1281	87.8
Delayed implementation	1.4 (-16.3 – 19.2)	0.875	0.8 (0.1 – 1.5)	0.028	-79.5 (-106.1 – -53.0)	<0.001	-4.9 (-7.5 – -2.3)	<0.001	1216 1252	89.6
Weekly intervals	-7.7 (-58.3 – 42.9)	0.766	0.1 (-1.3 – 1.5)	0.881	29.9 (5.8 – 54.0)	0.015	-4.0 (-6.8 – -1.3)	0.004	2744 2788	73.3

	Announcement				Implementation				Model fit	
	Step		Slope		Step		Slope			
	Coefficient (95% CI)	P value	Coefficient (95% CI)	P value	Coefficient (95% CI)	P value	Coefficient (95% CI)	P value	AIC/ BIC	Adj. R^2
**SMOKING-RELATED ILLNESS: ALL**										
Closed prisons	-76.4<br>(-458.9 – 306.1)	0.695	-23.9<br>(-41.4 – -6.4)	0.007	-646.2<br>(-1,110.9 – -181.4)	0.006	16.5<br>(-13.6 – 46.7)	0.282	2271<br>2304	54.6
All prisons	-112.5<br>(-479.7 – 254.8)	0.548	-22.8<br>(-39.4 – -6.1)	0.007	-716.3<br>(-1,160.5 – -272.2)	0.007	-16.6<br>(-12.1 – 45.4)	0.257	2270<br>2303	54.8
Adjusted for crowding	-70.2<br>(-466.0 – 325.6)	0.728	-24.7<br>(-43.6 – -5.8)	0.011	-657.6<br>(-1,130.6 – 184.6)	0.006	16.2<br>(-14.5 – 47.0)	0.300	2273<br>2309	54.6
Delayed implementation	-33.7<br>(-377.7 – 310.3)	0.848	-26.7<br>(-41.0 – -12.4)	<0.001	-752.0<br>(-1,253.8 – -250.1)	0.003	33.8<br>(0.2 – 67.3)	0.049	2269<br>2301	56.7
Weekly intervals	-63.5<br>(-431.4 – 304.4)	0.735	-11.7<br>(-21.3 – -2.1)	0.017	-752.1<br>(-1,256.3 – -247.9)	0.003	10.3<br>(-5.2 – 25.8)	0.192	4472<br>4809	35.8
**SMOKING-RELATED ILLNESS: RESPIRATORY**										
Closed prisons	65.7<br>(-130.4 – 261.9)	0.511	-4.3<br>(-13.0 – 4.4)	0.330	-485.9<br>(-746.7 – -225.1)	<0.001	-11.0<br>(-24.7 – 2.7)	0.114	2131<br>2170	61.4
All prisons	80.6<br>(-172.8 – 334.1)	0.533	-3.7<br>(-14.7 – 7.2)	0.506	-552.9<br>(-819.4 – -286.4)	<0.001	-10.0<br>(-26.0 – 6.0)	0.221	2147<br>2177	54.8
Adjusted for crowding	77.1<br>(-130.5 – 284.6)	0.467	-5.8<br>(-15.5 – 3.9)	0.243	-510.6<br>(-772.2 – -249.0)	<0.001	-11.9<br>(-25.9 – 2.2)	0.098	2132<br>2174	61.6
Delayed implementation	128.0<br>(-73.4 – 329.3)	0.213	-7.3<br>(-15.3 – 0.6)	0.071	-631.6<br>(-925.9 – -337.3)	<0.001	3.6<br>(-13.9 – 21.2)	0.687	2132<br>2168	60.4
Weekly intervals	109.7<br>(-129.6 – 348.8)	0.369	-2.4<br>(-8.3 – 3.6)	0.436	-556.0<br>(-859.1 – -252.9)	<0.001	-4.0<br>(-12.7 – 4.8)	0.375	4512<br>4549	43.7
**SMOKING-RELATED ILLNESS: CARDIOVASCULAR**										
Closed prisons	-105.6<br>(-191.4 – -19.8)	0.016	1.3<br>(-1.9 – 4.5)	0.418	-49.6<br>(-170.8 – 71.6)	0.422	-2.2<br>(-9.4 – 5.1)	0.561	1769<br>1790	12.1
All prisons	-105.0<br>(-185.7 – -24.4)	0.011	1.0<br>(-2.1 – 4.1)	0.529	-41.1<br>(-152.8 – 70.6)	0.471	-2.1<br>(-8.9 – 4.7)	0.546	1766<br>1790	12.1
Adjusted for crowding	-107.7<br>(-196.6 – -18.7)	0.018	1.7<br>(-1.8 – 5.1)	0.342	-44.9<br>(-164.6 – 74.9)	0.463	-2.0<br>(-9.2 – 5.1)	0.581	1771<br>1795	12.3
Delayed implementation	-92.3<br>(-178.5 – -6.2)	0.036	0.3<br>(-2.8 – 3.3)	0.865	-6.0<br>(-132.8 – 120.8)	0.926	-2.5<br>(-10.9 – 5.8)	0.551	1771<br>1792	11.3
Weekly intervals	-99.5<br>(-173.7 – -25.2)	0.009	0.6<br>(-1.0 – 2.2)	0.438	-45.1<br>(-134.1 – 43.9)	0.321	-1.0<br>(-3.9 – 1.8)	0.473	3686<br>3723	14.7

	Announcement				Implementation				Model fit	
	Step		Slope		Step		Slope			
	Coefficient (95% CI)	P value	Coefficient (95% CI)	P value	Coefficient (95% CI)	P value	Coefficient (95% CI)	P value	AIC/ BIC	Adj. R^2
**SMOKING-RELATED ILLNESS: GASTROINTESTINAL**										
Closed prisons	-73.9 (-303.7 – 156.0)	0.529	-21.0 (-32.3 – -9.7)	<0.001	-137.3 (-507.6 – 233.1)	0.468	30.2 (8.6 – 51.7)	0.006	2122 2143	27.6
All prisons	-106.5 (-330.6 – 117.7)	0.352	-20.5 (-31.1 – -9.8)	<0.001	-164.1 (-523.1 – 194.9)	0.370	30.1 (8.7 – 51.5)	0.006	2116 2137	28.9
Adjusted for crowding	-66.9 (-294.0 – 160.1)	0.563	-22.1 (-34.0 – -10.3)	<0.001	-153.0 (-524.8 – 218.8)	0.420	29.7 (7.9 – 51.5)	0.008	2124 2148	27.8
Delayed implementation	-76.7 (-294.8 – 141.4)	0.491	-20.8 (-30.1 – - 11.4)	<0.001	-113.9 (-568.8 – 341.0)	0.624	33.7 (5.8 – 61.7)	0.018	2122 2143	28.0
Weekly intervals	-82.7 (-323.3 – 157.8)	0.500	-10.3 (-16.5 – -4.2)	0.001	-138.8 (-478.3 – 200.8)	0.423	15.5 (5.0 – 25.9)	0.004	4447 4473	15.6
**SMOKING-RELATED ILLNESS: SENSORY**										
Closed prisons	1.4 (0.1 – 2.6)	0.032	-0.1 (-0.2 – 0.0)	0.002	-0.8 (-3.5 – 1.8)	0.541	0.2 (0.0 – 0.3)	0.017	670 697	37.8
All prisons	1.2 (0.0 – 2.4)	0.053	-0.1 (-0.2 – 0.0)	0.004	-0.9 (-3.7 – 1.8)	0.503	0.2 (0.0 – 0.3)	0.034	666 693	37.8
Adjusted for crowding	1.4 (0.2 – 2.7)	0.023	-0.1 (-0.2 – 0.0)	0.001	-1.0 (-3.8 - 1.8)	0.483	0.2 (0.0 – 0.3)	0.020	672 702	38.1
Delayed implementation	1.4 (0.2 – 2.7)	0.025	-0.1 (-0.2 – 0.0)	0.001	-0.2 (-2.7 – 2.4)	0.892	0.2 (0.0 – 0.3)	0.016	670 697	37.8
Weekly intervals	1.3 (0.1 – 2.6)	0.038	0.0 (-0.1 – 0.0)	0.001	-1.0 (-3.4 – 1.3)	0.399	0.1 (0.0 – 0.2)	0.011	1513 1547	37.5
**MENTAL HEALTH: ALL ANTI-DEPRESSANTS**										
Closed prisons	-119.7 (-270.2 – 30.7)	0.119	-2.9 (-11.5 – 5.6)	0.502	151.7 (-114.2 – 417.5)	0.263	0.5 (-12.6 – 13.6)	0.940	2145 2172	81.2
All prisons	-113.1 (-259.9 – 33.7)	0.131	-3.8 (-12.2 – 4.6)	0.374	150.6 (-118.1 – 419.3)	0.272	1.0 (-12.8 – 14.8)	0.882	2137 2164	82.0
Adjusted for crowding	-100.1 (-272.3 – 72.0)	0.254	-4.0 (-13.3 – 5.3)	0.396	114.8 (-163.1 – 392.6)	0.418	0.7 (-12.4 – 13.8)	0.912	2146 2176	81.2
Delayed implementation	-133.8 (-263.6 – -4.0)	0.043	-2.0 (-9.0 – 5.0)	0.577	158.2 (-122.3 – 438.6)	0.269	-2.2 (-19.6 – 15.3)	0.809	2146 2172	81.1
Weekly intervals	-107.1 (-265.8 – 51.6)	0.186	-1.7 (-6.2 – 2.7)	0.440	198.0 (-55.1 – 451.2)	0.125	-0.5 (-5.6 – 4.7)	0.861	4490 4523	70.9

	Announcement				Implementation				Model fit	
	Step		Slope		Step		Slope			
	Coefficient (95% CI)	P value	Coefficient (95% CI)	P value	Coefficient (95% CI)	P value	Coefficient (95% CI)	P value	AIC/ BIC	Adj. R^2
**MENTAL HEALTH: SSRI ONLY**										
Closed prisons	-121.3 (-171.9 – -70.7)	<0.001	-3.1 (-5.7 – -0.5)	0.020	18.2 (-52.0 – 88.4)	0.611	3.2 (-0.1 – 6.4)	0.054	1729 1753	70.4
All prisons	-118.7 (-167.4 – -70.0)	<0.001	-3.3 (-5.8 – -0.8)	0.011	18.8 (-50.0 – 87.6)	0.592	3.0 (-0.2 – 6.2)	0.068	1722 1746	71.7
Adjusted for crowding	-116.3 (172.2 – -60.5)	<0.001	-3.4 (-6.2 – -0.6)	0.018	9.3 (-64.0 – 82.6)	0.804	3.2 (-0.1 – 6.5)	0.059	1730 1757	70.6
Delayed implementation	-123.0 (-166.1 – -79.9)	<0.001	-3.0 (-4.9 – -1.0)	0.003	32.8 (-40.3 – 105.9)	0.379	2.5 (-2.5 – 7.1)	0.278	1729 1753	70.5
Weekly intervals	-118.3 (-174.5 – -62.2)	<0.001	-1.6 (-3.1 – -0.2)	0.028	29.9 (-46.0 – 105.7)	0.440	1.5 (-0.2 – 3.1)	0.080	3635 3668	59.2
**MENTAL HEALTH: HYPNOTICS/ANXIOLYTICS [post-protocol analysis]**										
Closed prisons	8.8 (-79.0 – 96.7)	0.844	-0.3 (-4.0 – 3.4)	0.877	-17.1 (-138.0 – 103.8)	0.782	1.4 (-6.0 – 8.8)	0.708	1862 1886	5.8
All prisons	8.1 (-77.3 – 93.4)	0.853	-0.4 (-3.9 – 3.2)	0.842	-16.7 (133.5 – 100.2)	0.780	1.4 (-5.7 – 8.5)	0.700	1854 1878	6.3
Adjusted for crowding	25.4 (-66.8 – 117.5)	0.590	-1.8 (-6.0 – 2.5)	0.413	-45.8 (-165.7 – 74.0)	0.454	1.2 (-6.1 – 8.5)	0.750	1862 1889	7.3
Delayed implementation	-3.1 (-90.7 – 84.6)	0.945	0.6 (-2.9 – 4.1)	0.729	-87.4 (-236.5 – 61.6)	0.250	3.9 (-5.4 – 13.3)	0.411	1861 1885	6.9
Weekly intervals	3.5 (-71.2 – 78.3)	0.926	0.1 (-1.4 – 1.5)	0.911	-36.9 (-136.7 – 62.9)	0.469	0.7 (-2.0 – 3.4)	0.606	4002 4039	6.5
**CONTROL: ANTI-EPILEPTICS**										
Closed prisons	-19.0 (-94.8 – 56.8)	0.623	2.1 (-1.3 – 5.5)	0.230	-70.9 (-184.8 – 43.1)	0.223	-0.7 (-6.6 – 5.2)	0.820	1752 1782	45.7
All prisons	-30.0 (-104.1 – 44.2)	0.428	2.4 (-1.0 – 5.7)	0.166	-80.0 (-193.2 – 33.1)	0.166	-1.1 (-7.0 – 4.8)	0.715	1746 1776	49.5
Adjusted for crowding	-12.0 (-87.9 – 63.9)	0.757	1.7 (-1.8 – 5.2)	0.333	-78.0 (-190.6 – 34.6)	0.175	-0.7 (-6.5 – 5.2)	0.826	1754 1787	45.9
Delayed implementation	-15.9 (-92.1 – 60.3)	0.683	1.9 (-1.5 – 5.3)	0.275	-101.8 (-209.4 – 5.7)	0.063	1.7 (-5.0 – 8.4)	0.610	1751 1781	46.2
Weekly intervals	13.9 (-66.7 – 94.5)	0.735	0.5 (-1.4 – 2.5)	0.591	-32.1 (-119.6 – 55.5)	0.473	-0.4 (-2.7 – 2.0)	0.767	3722 3759	28.9

Section S7. Relative effect estimates, expressed as percentage change in fortnightly rate of dispensing per 1000 persons in custody: primary analysis (closed prisons)

	Announcement		Implementation	
	**Step**	**Slope**	**Step**	**Slope**
	Percentage change (95% CI)	Percentage change (95% CI)	Percentage change (95% CI)	Percentage change (95% CI)
All nicotine dependence	19.7 (-150.6 – 111.2)	1.2 (-2.7 – 5.1)	43.5 (36.2 – 50.7)	-0.1 (-0.9 – 0.8)
Nicotine replacement therapy	-26.2 (-157.4 – 105.0)	1.5 (-2.4 – 5.3)	42.3 (34.1 – 50.4)	0.1 (-0.6 – 0.9)
Other medications for nicotine dependence (varenicline/bupropion)	-5.8 (-55.1 – 43.5)	0.1 (-2.5 – 2.7)	27.0 (11.2 – 42.9)	-5.1 (-8.1 – -2.1)
All smoking-related illness	-1.0 (-5.8 – 3.9)	-0.3 (-0.5 – -0.1)	-8.6 (-14.9 – -2.4)	0.2 (-0.2 – 0.6)
Smoking-related illness: respiratory	1.4 (-2.8 – 5.7)	-0.1 (-0.3 – 0.1)	-10.9 (-16.7 – -5.0)	-0.2 (-0.6 – 0.1)
Smoking-related illness: cardiovascular	-43.9 (-79.5 – -8.2)	0.5 (-0.8 – 1.9)	-28.3 (-97.6 – 40.9)	-1.3 (-5.4 – 2.9)
Smoking-related illness: gastrointestinal	-2.4 (-10.0 – 5.1)	-0.7 (-1.1 – -0.3)	-4.8 (-17.9 – 8.2)	1.1 (0.3 – 1.8)
Smoking-related illness: sensory	23.3 (1.7 – 43.3)	-1.7 (-3.3 – 0.0)	-19.0 (-83.3 – 42.9)	4.8 (0.0 – 7.1)
Mental health: all anti-depressants	-2.8 (-6.4 – 0.7)	-0.1 (-0.3 – 0.1)	2.9 (-2.2 – 7.9)	0.0 (-0.2 – 0.3)
Mental health: SSRI only	-12.1 (-17.1 – -7.0)	-0.3 (-0.6 – 0.0)	1.7 (-5.0 – 8.5)	0.3 (0.0 - 0.6)
Mental health: hypnotics/anxiolytics	1.0 (-8.8 – 10.8)	0.0 (-0.4 – 0.4)	-2.1 (-17.2 – 12.9)	0.2 (-0.7 – 1.1)
Control: anti-epileptics	-2.4 (-11.8 – 7.1)	0.3 (-0.2 – 0.7)	-7.3 (-18.9 – 4.4)	-0.1 (-0.7 – 0.5)

Section S8. Results of indicator saturation analyses

Indicator saturation analyses allow the identification of multiple step or slope changes by testing for breaks at every point in the time series and removing all but the significant breaks (at the level specified), using general-to-specific model selection 17. We built indicator saturation models using the *gets* package in R version 3.6.3, using time series properties identified from the ACF/PACF plots and final set of Stata models. Models for all outcomes were specified to test for step or slope changes, using a p value set at p<0.0001. The table below shows the date and magnitude of step and slope changes identified as significant under these parameters. Note that the inclusion of slope variables influences the validity of standard errors so confidence intervals should be interpreted with caution.

	**Step changes**			**Slope changes**			**Model properties**		
	Date	Coefficient (95% CI)	P value	Date	Coefficient (95% CI)	P value	Ljung Box AR	Ljung Box ARCH	Adj. R^2
**All nicotine dependence**	02-12-2018	-6034.2 (-6981.7 – -5086.7)	<0.001	11-08-2019	-1357.0 (-1788.9 – -925.2)	<0.001	0.926	0.336	94.4
	13-01-2019	6091.4 (4933.5 – 7249.4)	<0.001	06-10-2019	1438.2 (934.6 – 1941.8)	<0.001			
	24-02-2019	4975.3 (3849.0 – 6101.6)	<0.001	-	-	-			
	24-03-2019	6081.1 (5033.4 – 7128.8)	<0.001	-	-	-			
	07-04-2019	-2456.4 (-3528.7 – -1384.1)	<0.001	-	-	-			
	21-04-2019	6095.1 (4989.8 – 7200.5)	<0.001	-	-	-			
	11-08-2019	3564.8 (2358.9 – 4770.7)	<0.001	-	-	-			
**Nicotine replacement therapy**	02-12-2018	-4800.4 (-5651.4 – -3949.4)	<0.001	16-12-2018	1174.0 (1026.8 – 1321.2)	<0.001	0.746	0.505	93.9
	13-01-2019	6362.8 (5198.7 – 7527.0)	<0.001	30-12-2018	-3305.8 (-3701.1 – -2910.5)	<0.001			
	24-02-2019	4963.8 (3819.3 – 6108.3)	<0.001	19-05-2019	2467.5 (2068.5 – 2866.5)	<0.001			
	24-03-2019	5936.0 (4879.4 – 6992.6)	<0.001	-	-	-			
	07-04-2019	-2761.5 (-3833.8 – -1689.2)	<0.001	-	-	-			

	21-04-2019	6019.7 (4976.3 – 7207.2)	<0.001	-	-	-			
	11-08-2019	-2107.1 (-2847.2 – -1367.1)	<0.001	-	-	-			
**Other medications for nicotine dependence (varenicline/bupropion)**	16-12-2018	-94.3 (-124.9 – -63.6)	<0.001	-	-	-	0.755	0.832	87.4
	30-12-2018	83.8 (51.4 – 116.1)	<0.001	-	-	-			
**Smoking-related illness - all**	21-12-2014	-8022.4 (-9909.7 – -6135.2)	<0.001	06-12-2015	791.3 (340.7 – 1241.9)	<0.001	0.502	0.168	65.3
	04-01-2015	7975.4 (6055.5 - 9895.4)	<0.001	31-01-2016	-809.8 (-1260.1 – -359.6)	<0.001			
	06-12-2015	-2913.1 (-4706.9 – -1119.3)	0.002	-	-	-			
	16-12-2018	-681.0 (-1023.8 – -338.2)	<0.001	-	-	-			
**Respiratory**	16-12-2018	-921.3 (-1101.5 – -741.1)	<0.001	-	-	-	0.558	0.173	65.0
**Cardiovascular**	-	-	-	-	-	-	-	-	-
**Gastrointestinal**	-	-	-	24-05-2015	19.9 (5.2 – 13.6)	<0.001	0.694	0.122	27.6
				03-07-2016	-24.7 (-31.5 – -17.8)	<0.001			
**Sensory**	09-04-2017	9.4 (5.2 - 13.6)	<0.001	-	-	-	0.409	0.321	45.4
	27-08-2017	-19.5 (-23.9 – -15.1)	<0.001	-	-	-			
	20-05-2018	-3.1 (-5.1 – -1.1)	0.003	-	-	-			
**Mental health – all antidepressants**	20-12-2015	-1611.7 (-2265.0 – -958.3)	<0.001	-	-	-	0.987	0.304	85.3
	03-01-2016	1541.8 (881.0 – 2202.6)	<0.001	-	-	-			
**Mental health - SSRI**	20-12-2015	-298.9 (-442.4 – -155.5)	<0.001	-	-	-	0.951	0.802	84.8
	03-01-2016	318.8 (177.3 – 460.2)	<0.001	-	-	-			

**Mental health – hypnotics/anxiolytics**	-	-	-	-	-	-	-	-	-
**Control – anti-epileptics**	14-02-2016	62.4 (20.9 – 103.9)	0.004	-	-	-	0.532	0.850	63.0
	08-10-2017	69.3 (11.5 – 127.0)	0.020	-	-	-			
